# Sharing space between native and invasive small mammals: Study of commensal communities in Senegal

**DOI:** 10.1002/ece3.10539

**Published:** 2023-09-20

**Authors:** Laurent Granjon, Emanuelle Artige, Khalilou Bâ, Carine Brouat, Ambroise Dalecky, Christophe Diagne, Mamoudou Diallo, Odile Fossati‐Gaschignard, Philippe Gauthier, Mamadou Kane, Laëtitia Husse, Youssoupha Niang, Sylvain Piry, Nathalie Sarr, Aliou Sow, Jean‐Marc Duplantier

**Affiliations:** ^1^ CBGP, IRD, INRAE, CIRAD, Montpellier SupAgro, Univ Montpellier Montpellier France; ^2^ BIOPASS, CBGP‐IRD, ISRA, UCAD, CIRAD Dakar Senegal; ^3^ IRD, Aix Marseille Univ, LPED Marseille France

**Keywords:** community ecology, co‐occurrence, rodents, shrews, West Africa

## Abstract

Urbanization processes are taking place at a very high rate, especially in Africa. At the same time, a number of small mammal species, be they native or invasive, take advantage of human‐induced habitat modifications. They represent commensal communities of organisms that cause a number of inconveniences to humans, including potential reservoirs of zoonotic diseases. We studied via live trapping and habitat characterization such commensal small mammal communities in small villages to large cities of Senegal, to try to understand how the species share this particular space. Seven major species were recorded, with exotic invasive house mice (*Mus musculus*) and black rats (*Rattus rattus*) dominating in numbers. The shrew *Crocidura olivieri* appeared as the main and more widespread native species, while native rodent species (*Mastomys natalensis*, *M. erythroleucus*, *Arvicanthis niloticus* and *Praomys daltoni*) were less abundant and/or more localized. Habitat preferences, compared between species in terms of room types and characteristics, showed differences among house mice, black rats and *M. natalensis* especially. Niche (habitat component) breadth and overlap were measured. Among invasive species, the house mouse showed a larger niche breadth than the black rat, and overall, all species displayed high overlap values. Co‐occurrence patterns were studied at the global and local scales. The latter show cases of aggregation (between the black rat and native species, for instance) and of segregation (as between the house mouse and the black rat in Tambacounda, or between the black rat and *M. natalensis* in Kédougou). While updating information on commensal small mammal distribution in Senegal, a country submitted to a dynamic process of invasion by the black rat and the house mouse, we bring original information on how species occupy and share the commensal space, and make predictions on the evolution of these communities in a period of ever‐accelerating global changes.

## Graphical abstract

We present and analyse data on the distribution of commensal small mammals obtained via intensive trapping operation and habitat description in a series of localities in Senegal, with the aim to: (i) describe the community of small mammals living in contact with humans and evaluate the respective share that native and invasive species occupy with this community; (ii) better understand the factors that govern species distribution and co‐occurrence of species using a series of analyses using habitat characteristics gathered at the moment of captures, in view of a better understanding of patterns and processes at work in a group of organisms of particular concern regarding their interactions with humans.

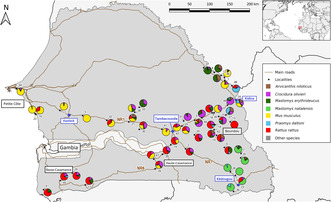

## 1 INTRODUCTION

Urbanization in developing countries has for long been identified as a major process with multiple consequences at the global scale (Cohen, 2006; Henderson & Turner, 2020). This process comprises both the emergence of megacities and the rapid growth of small and medium cities (Cohen, 2006; United Nations, Department of Economic and Social Affairs, Population Division, 2018). As a result, urban areas are projected to house 60% of people globally by 2030, with almost 90% of this growth taking place in Asia and Africa (un.org). The corresponding rise of the “indoor biome” raises new eco‐evolutionary questions regarding species, and species communities, associated with this expanding environment (Martin et al., 2015; Hulme‐Beaman et al., 2016). Long composed mainly of native species, these communities (like others from more natural biomes) have been progressively colonized by introduced species benefitting from human‐caused global changes (Vitousek et al., 1997).

Among other groups of living organisms, rodents comprise species that are especially prone to take advantage of the modification of habitats by human activities, being core species in an urbanization context (Capizzi et al., 2014; Rothenburger et al., 2017). Their impacts are diverse and multidimensional, including notably biodiversity loss (Doherty et al., 2016), threats to food security (Singleton et al., 2021), disease transmission (Han et al., 2015), economic burdens (Diagne et al., 2023; Dossou et al., 2020) and societal decay (Colombe et al., 2019). Some of these rodents are well‐known as major invasive alien species (hereafter “invasive rodents”) worldwide (Capizzi et al., 2014; Lowe et al., 2000). This is the case of *Rattus rattus*, the black rat, and *Mus musculus*, the house mouse, which are both listed among “100 of the world's worst invasive species” on the planet (Lowe et al., 2000). The ongoing expansion of those invasive rodents in several parts of the world (see, for instance, Dalecky et al., 2015; Yu et al., 2022; Zeng et al., 2018) leads to multispecific assemblages of small mammals (mainly rodents) that combine invasive and native species in a variety of ecological and evolutionary contexts. One of these contexts is represented by commensalism in anthropogenic environments, where the species concerned literally “live within houses”, in close proximity to humans (Hulme‐Beaman et al., 2016). There, despite ever‐increasing studies on the distribution (including invasion history of invasive small mammals), impacts and dynamics of individual species over space and time, the multispecific assemblages of these rodent‐dominated communities have rarely been studied from a community ecology perspective.

Species sampling in anthropogenic habitats is often complicated because it involves going into people's homes or industrial or commercial buildings. Moreover, even if rodent communities in such habitats are often depauperate, diversity is generally not taken into account because one focuses on a (or a pair of) target species in relation to specific questions raised by it/them. This is the case in the review by Feng and Himsworth (2014) on *R. norvegicus* and *R. rattus*, in the studies focusing on the impact of urban characteristics on the genetic structure of rodent populations in different cities (*R. norvegicus* in American cities: Combs et al., 2018; *M. musculus* in Dakar (Senegal): Stragier et al., 2020), or in experiments on species cohabitation and interspecific competition involving *M. musculus* in SW Argentina (Castillo et al., 2003; Gomez et al., 2008). However, some studies have already considered more complete communities. For instance, Panti‐May et al. (2012) measured data on abundance, population and habitat use parameters of *M. musculus* and *R. rattus* among their native counterparts in households in a rural area of Mexico as part of a study on zoonotic disease transmission. Masi et al. (2010) evaluated the respective importance of socioeconomic and environmental risk factors for urban rodent (including *R. rattus*, *R. norvegicus* and *M. musculus*) infestation in Sao Paulo, Brazil. Cavia et al. (2009) analysed the relation between rodent community composition and diversity and the landscape structure in the city of Buenos Aires, showing a clear trend of habitat partitioning among invasive *R. rattus, R. norvegicus* and/or *M. musculus* (dominant in parklands, shantytowns or industrial–residential neighbourhoods) and native species (not only dominant in a natural reserve but also present in parklands). In Africa, Olaseha et al. (1994) presented general considerations on the importance of housing and sanitation on the presence of rats (*R. rattus* and *R. norvegicus*) and mice (*M. musculus*) based on questionnaires completed by interviews in towns and villages of a rural area in south‐western Nigeria. Demby et al. (2001) followed by Fichet‐Calvet et al. (2005, 2009) provided information on small mammal distribution in urban as well as in rural areas of Guinea, in relation to Lassa virus distribution and prevalence. Taylor et al. (2008) gave a few elements of urban distribution of rodents in Durban (South Africa) in a small mammal community largely dominated by *R. norvegicus*. Monadjem et al. (2011) compared movement patterns and possible interactions of *Mastomys natalensis* (a native rodent species) and *R. rattus* in distant sites of Tanzania, Malawi and Namibia using telemetry and Rhodamine B marker. In the capital city of Niger, Niamey, Garba et al. (2014) analysed the distribution of native and invasive rodents in a series of sites corresponding to habitation districts, cultivated gardens and industrial zones. They showed the dominance of the native *M. natalensis* over the invasive *R. rattus* and *M. musculus* and spatial segregation between them, which they interpreted as the result of an ongoing native‐to‐invasive species turn over. Hima et al. (2019) assembled an important dataset on commensal small mammal distribution in a series of localities along the Benin‐Niger “corridor”, between Cotonou and Niamey. They showed the dominance of either invasive *R. rattus* in Cotonou (see also Houemenou et al., 2014) or native *M. natalensis* in Niamey, with segregation patterns between *Rattus* spp. and *M. natalensis*, and a very regular and important presence of *Crocidura* spp. (incl. *C. olivieri*), especially at lower latitudes. None of these studies has nonetheless addressed in detail the co‐distribution and coexistence of a set of species (both native and invasive) belonging to a whole small mammal community in human‐made environments, especially at a fine spatial scale. At best, they considered co‐occurrence patterns at the scale of a country, a region or a whole city, but never at the level of the housing units or the buildings, where inter‐individual (be they intra‐ or interspecific) interactions actually occur. Yet it is precisely at this fine scale that the ecological interactions take place which probably determine the trajectory of the communities in terms of their distribution in space and time.

Niche/resource partitioning represents a way to manage coexistence among competing species within habitats (Chesson, 2000; Pianka, 1973). Indeed, the complex and interactive effects of species niche overlap, niche breadth and environmental heterogeneity on species co‐occurrence patterns have been highlighted repeatedly (see a synthesis in Bar‐Massada, 2015). In the particular case of commensal small‐mammal communities, information on and analyses of species co‐distribution and co‐existence, habitat partitioning (if any) and interspecific interactions between species (including invasive ones) are lacking, being, however, of paramount importance to better understand: (i) the way invasive species spread at the microhabitat scale; (ii) the consequences of this spread on the distribution of native species at the microhabitat scale; and (iii) the actual associations between species likely to represent zoonotic disease reservoirs at the very contact with humans.

In Africa, both *R. rattus* and *M. musculus* colonized most countries via boats of European or Arab settlers, often centuries ago (Happold, 2013). Long confined to coastal areas and larger cities, they have been spreading continuously over inland areas thanks to the development of infrastructures and associated human exchanges (e.g. movements of goods and people) that accompany the ongoing urbanization of rural areas. This is the case in Senegal (West Africa) where both *R. rattus* and *M. musculus* have experienced recent range expansion eastward from the western Atlantic coastal areas (Dalecky et al., 2015; Duplantier et al., 1991; Konečný et al., 2013). Being exclusively commensal in this country, they encounter native species that inhabit human settlements, leading to inevitable interactions that ultimately determine the patterns of cohabitation between them. To describe this coexistence and try to understand the underlying interactions, we sampled communities of commensal small mammals from localities of various sizes within the southern half of Senegal (corresponding to the distribution range of *R. rattus* in the country) within a 3‐year time period. The sites sampled were widely invaded by the black rat and/or the domestic mouse, which most of the time cohabited with a wide spectrum of native rodent and shrew species. We aim to provide novel insights from the following questions: (i) which invasive and native species compose the small mammal community across the different localities targeted? (ii) what are the preferred habitat types and ecological niches of each of these species? and (iii) do these species show particular interspecific associations (segregation or aggregation) globally and/or locally? To answer these questions, we investigated here the composition, geographic distribution, micro‐habitat use, ecological niche breadth and overlap and species co‐occurrence within the target small mammal community at various spatial scales.

## 2 MATERIALS AND METHODS

Detailed trapping data are presented in Granjon et al. (2021) with, among others, information on the associated variables and capture results of each of the 13,283 trapnights that yielded the dataset analysed here.

### 2.1 Study area

**FIGURE 1:**
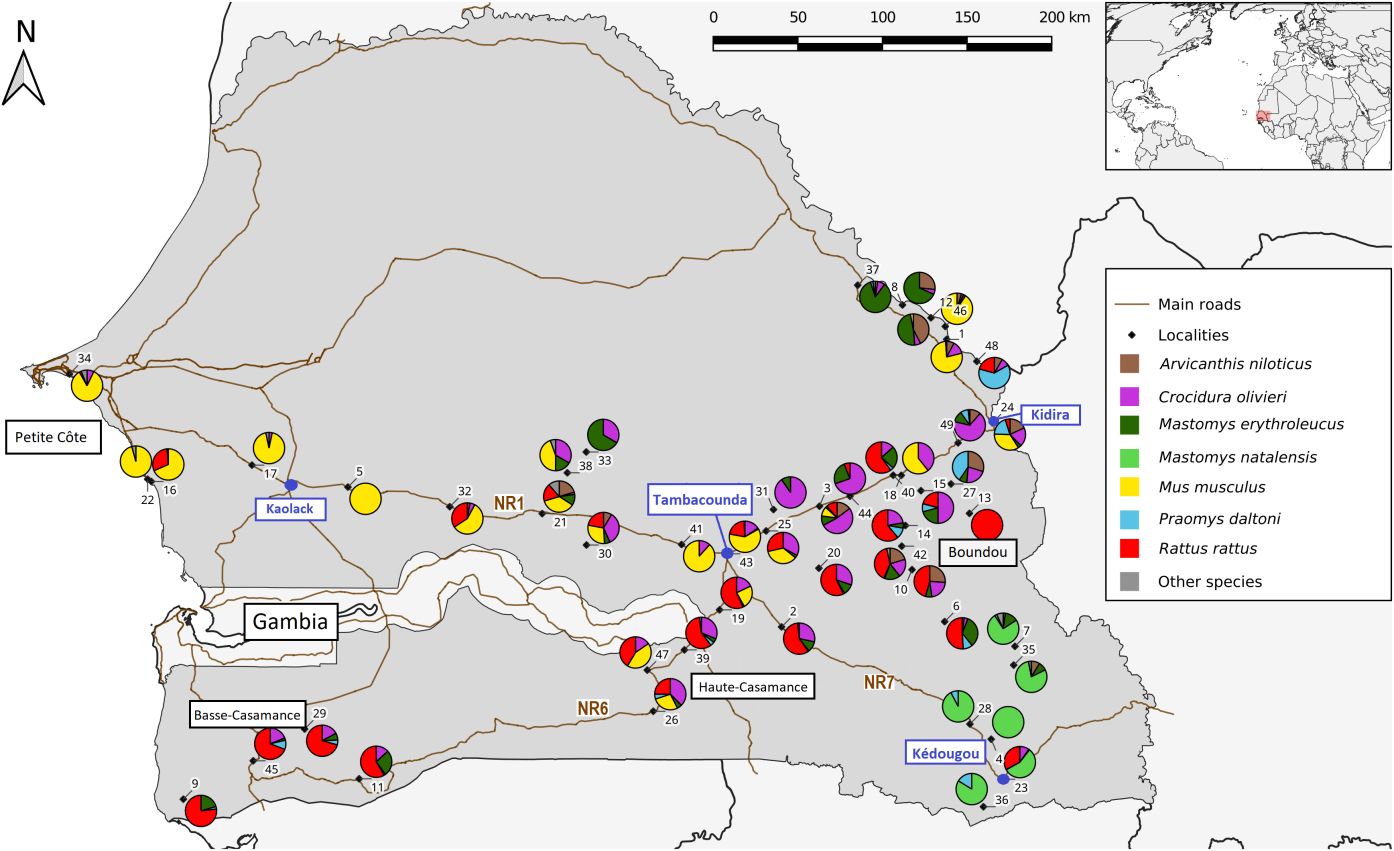
Relative frequencies of the small mammals caught in each of the 49 localities sampled. 1: Bakel, 2: Badi Nieriko, 3: Bala, 4: Bantako, 5: Birkelane, 6: Boutougoufara, 7: Bransan, 8: Dembankane, 9: Diakene‐Wolof, 10: Dianke Makha, 11: Diattacounda, 12: Diawara, 13: Dide Gassama, 14: Dieylany, 15: Doulouyabe, 16: Fadiouth, 17: Gandiaye, 18: Goudiry, 19: Gouloumbou, 20: Goumbayel, 21: Ida Seco, 22: Joal, 23: Kedougou, 24: Kidira, 25: Kothiary, 26: Kounkane, 27: Koussan, 28: Mako, 29: Marsassoum, 30: Mereto, 31: Ndiobene, 32: Niahene, 33: Panal, 34: Rufisque, 35: Sabodala, 36: Segou, 37: Seme, 38: Sil, 39: Sinthian Koundara, 40: Sinthiou Doube, 41: Sinthiou Maleme, 42: Soutouta, 43: Tambacounda, 44: Talibadji, 45: Tobor, 46: Tuabou, 47: Velingara, 48: Yafera and 49: Youppe Hamady (see Table 1 for geographic coordinates and home area of each locality). Modified from fig. 5 in Granjon et al. (2021). Country map retrieved from GADM 3.6 (https://gadm.org/index.html) and roads from OpenStreetMap (http://download.geofabrik.de/africa/senegal‐and‐gambia‐latest‐free.shp.zip). NR1, NR6 and NR7: National Roads 1, 6 and 7.

Forty‐nine localities were sampled between May 2012 and September 2015 throughout the southern half of Senegal (between 12.40° and 15.20° N, and 12° and 17.30° W). They are listed in Table 1 with their geographic coordinates (see also Figure 1). In terms of human population, they range between a few hundreds to around 500,000 inhabitants (Rufisque) and accordingly, they were sampled during periods ranging between 2 and 21 days. Inter‐locality distances range between 3.5 km (between Joal and Fadiouth island, linked by a pedestrian bridge) and 601.5 km (between Rufisque and Kédougou), with a mean value of 220 km. Given what we know of the limited dispersal capacities of the small mammal species concerned, we consider these localities as independent from each other. They can be grouped into nine areas/localities as follows: North of the Gambia, the “*Petite Côte*” area along the Atlantic coast south of Dakar and the “*Kaolack‐Tambacounda*” axis along National Road (NR) 1; South of the Gambia along NR 6, “*Basse Casamance*” in the West and “*Haute Casamance*” in the East; from the main city of *Tambacounda*, lying at the crossroad of National Roads 1, 6 and 7, the “*Tambacounda‐Kidira”* axis along NR1 to the Senegal‐Mali border; South of this last axis, the relatively landlocked “*Boundou”* area, and along the Senegal‐Mauritania border, the “*Bakel”* area; and in the extreme south‐east, the “*Kédougou”* area. These areas are delimited in Appendix 1 with included localities (see also Table 1).

**TABLE 1:** List of localities sampled with their geographic coordinates (in decimal degrees), trapping effort (trapnight number) and numbers of specimens of small mammals captured (Tamba = Tambacounda).

Locality	Lat_N	Lon_W	Area	Trapnight number	*Arvicanthis niloticus*	*Atelerix albiventris*	*Cricetomys gambianus*	*Crocidura olivieri*	*Crocidura* sp.	*Gerbilliscus gambianus*	*Mastomys erythroleucus*	*Mastomys natalensis*	*Mastomys* sp.	*Mus* (*Nannomys*) sp.	*Mus musculus*	*Mus* sp.	*Praomys daltoni*	*Rattus rattus*	*Steatomys* sp.
Badi Nieriko	13.377	13.376	Boundou	379	0	0	0	36	2	0	14	0	0	0	0	0	1	74	0
Bakel	14.904	12.458	Bakel	345	7	0	0	12	1	0	0	0	0	0	70	0	0	0	0
Bala	14.020	13.166	Tamba‐Kidira	237	9	0	0	32	0	0	6	0	0	0	6	0	1	7	0
Bantako	12.767	12.239	Kedougou	150	0	0	0	0	0	0	0	47	0	0	0	0	0	0	0
Birkelane	14.130	15.750	Kaolack‐Tamba	110	0	0	0	0	0	0	0	0	0	0	32	0	0	0	0
Boutougoufara	13.398	12.486	Boundou	462	2	0	0	4	0	0	34	0	0	0	0	0	10	52	0
Bransan	13.262	12.104	Kedougou	145	1	0	0	0	3	0	6	33	0	0	0	0	1	0	0
Dembankane	15.091	12.700	Bakel	200	16	0	0	3	0	0	41	0	0	0	0	0	0	0	0
Diakene‐Wolof	12.456	16.636	Basse Casamance	129	0	0	0	0	0	0	8	0	0	0	0	0	1	30	0
Dianke Makha	13.679	12.661	Boundou	154	12	0	0	10	0	0	3	0	0	0	0	0	0	21	0
Diattacounda	12.57	15.682	Basse Casamance	171	0	0	0	8	0	0	16	0	0	0	0	0	1	35	0
Diawara	15.021	12.544	Bakel	200	14	0	0	2	0	0	16	0	0	0	1	0	0	0	0
Dide Gassama	13.974	12.343	Tamba‐Kidira	176	0	0	0	0	0	0	0	0	0	0	0	0	0	30	0
Dieylany	13.913	12.695	Tamba‐Kidira	345	0	0	0	4	0	0	1	0	0	0	0	0	2	11	0
Doulouyabe	14.098	12.608	Tamba‐Kidira	225	0	0	0	12	0	0	5	0	0	0	0	0	2	5	0
Fadiouth	14.152	16.823	Petite‐Cote	299	0	0	0	0	0	0	0	0	0	0	52	1	0	23	0
Gandiaye	14.240	16.270	Kaolack‐Tamba	145	0	0	0	1	0	0	0	0	0	0	28	0	0	0	1
Goudiry	14.184	12.716	Tamba‐Kidira	244	0	0	0	22	0	0	0	0	0	0	34	0	0	0	0
Gouloumbou	13.470	13.717	Haute Casamance	221	0	0	0	12	0	0	0	0	0	0	16	0	1	38	0
Goumbayel	13.690	13.170	Boundou	158	0	0	0	19	0	0	7	0	0	0	0	0	1	36	0
Ida Seco	13.994	14.679	Kaolack‐Tamba	268	9	0	0	1	0	1	5	0	0	3	16	0	0	8	1
Joal	14.170	16.850	Petite‐Cote	372	0	0	4	0	0	0	0	0	0	0	89	0	0	0	0
Kedougou	12.554	12.179	Kedougou	1067	0	0	1	22	0	0	2	127	0	0	0	0	1	73	0
Kidira	14.457	12.212	Tamba‐Kidira	202	11	0	0	12	0	0	2	0	0	0	21	0	12	3	0
Kothiary	13.891	13.459	Tamba‐Kidira	248	0	0	0	23	0	0	2	0	0	0	25	0	0	20	0
Kounkane	14.932	14.075	Haute Casamance	168	0	0	0	15	0	0	2	0	0	0	11	0	2	10	0
Koussan	14.132	12.443	Tamba‐Kidira	284	11	0	0	8	0	0	3	0	0	0	0	0	15	0	0
Mako	12.850	12.353	Kedougou	140	0	0	0	0	0	0	0	48	0	0	0	0	4	0	0
Marsassoum	12.834	15.976	Basse Casamance	123	0	0	0	7	0	0	3	0	0	0	0	0	2	29	0
Mereto	13.818	14.438	Kaolack‐Tamba	322	9	0	0	36	0	0	6	0	0	0	30	0	0	23	0
Ndiobene	14.004	13.416	Tamba‐Kidira	208	0	0	0	28	0	0	3	0	0	0	0	0	0	0	0
Niahene	14.024	15.186	Kaolack‐Tamba	154	1	0	0	2	0	0	0	0	0	0	22	0	0	13	0
Panal	14.316	14.440	Kaolack‐Tamba	110	0	0	0	1	0	0	2	0	0	0	0	0	0	0	0
Rufisque	14.722	17.277	Petite‐Cote	685	0	0	6	7	0	0	0	0	0	0	83	0	0	2	0
Sabodala	13.162	12.112	Kedougou	150	3	0	0	0	0	0	3	27	1	0	0	0	0	0	0
Segou	12.408	12.285	Kedougou	143	0	0	0	0	0	0	0	26	0	0	0	0	5	0	0
Seme	15.198	12.944	Bakel	201	1	0	0	3	1	0	34	0	0	0	0	0	1	0	0
Sil	14.205	14.544	Kaolack‐Tamba	152	0	0	0	6	1	0	3	0	0	0	8	0	0	0	0
Sinthian Koundara	13.256	13.906	Haute Casamance	199	0	0	0	15	1	0	2	0	0	0	0	0	2	28	0
Sinthiou Doube	14.182	12.759	Tamba‐Kidira	141	0	0	0	4	0	0	7	0	0	0	0	0	1	18	0
Sinthiou Maleme	13.820	13.920	Kaolack‐Tamba	131	0	0	0	4	0	0	0	0	0	0	30	0	0	0	0
Soutouta	13.803	12.716	Boundou	398	24	0	0	22	3	0	19	0	1	0	0	0	1	47	0
Talibadji	14.072	12.997	Tamba‐Kidira	178	0	0	0	23	0	0	8	0	0	0	0	0	0	2	0
Tambacounda	13.769	13.667	Tambacounda	1957	0	1	1	79	0	0	6	0	0	0	307	1	0	108	1
Tobor	12.664	16.257	Basse Casamance	125	0	0	0	8	0	0	1	0	0	0	0	0	4	29	0
Tuabou	14.973	12.465	Bakel	122	2	0	0	1	0	0	1	0	0	0	39	0	0	0	0
Velingara	13.150	14.110	Haute Casamance	198	0	0	0	9	0	0	0	0	0	0	25	0	0	24	0
Yafera	14.785	12.294	Bakel	101	2	0	0	2	0	0	0	0	0	0	0	0	15	5	0
Youppe Hamady	14.351	12.402	Tamba‐Kidira	241	7	0	0	41	1	0	7	0	0	0	0	0	5	0	0
Total				13,283	141	1	12	556	13	1	278	308	2	3	945	2	91	804	3
Number of localities where present					18	1	4	39	8	1	33	6	2	1	21	2	24	29	3
Number of localities where dominant					0	0	0	7	0	0	4	6	0	0	16	0	2	14	0

### 2.2 Sampling scheme and protocols

Elements of the trapping procedures followed here have already been partly described by Dalecky et al. (2015), Diagne et al. (2021) and Granjon et al. (2021). The live traps used were of two types: locally made wire‐mesh live traps (8.5 × 8.5 × 26.5 cm) and Sherman (H.B. Sherman Traps, Inc., Tallahassee, Florida, USA) folding box traps (8 × 9 × 23 cm), which have proven to be complementary for the capture of different species present (Granjon et al., 2021). Traps were set inside housing or working units (e.g. dwelling houses, storehouses, shops and workshops) which potentially included inner yards and associated parts (e.g. exterior staircases and verandas). In each of these buildings, traps were set between one and three consecutive nights in different “rooms”. Most of the time, a trap of each type was placed in each room, usually on the floor and occasionally on furniture or even high up (wall tops, frame, etc.). We generally did not set traps in adjacent rooms to limit potential bait attraction from one room to the other. When the traps were initially set up, each room sampled was georeferenced (geographic coordinates GPS recorded with an accuracy of ±5 m). The rooms were classified as belonging to eight “room types,” namely bedrooms, granaries, food shops, kitchens, non‐food stores, outdoors, stock rooms and workshops. In the rooms, the presence or absence of food and the nature (materials) of the floor, walls and ceiling (see modalities in Figure 1 legend) were noted. This information represents markers of the type of habitat (traditional vs. modern) in which the small mammals studied live in contact with their human hosts. Traps were checked and then baited once a day with peanut butter spread on a slice of fresh onion.

Captured rodents were morphologically identified (following keys provided in Granjon & Duplantier, 2009), euthanized, then weighed to the nearest 0.5 g, sexed and dissected. When necessary, molecular data were generated to allow unambiguous species identification of rodents (following procedures described by Dobigny et al., 2011; Lecompte et al., 2005).

### 2.3 Data treatment

To test for the quality of sampling and the associated representativeness of the set of specimens caught relative to the actual communities sampled, diversity analyses were performed following the principles of Chao et al. (2014). Rarefaction and completeness curves corresponding to locality and area samples of specimens obtained were built based on data from Table 1, using Chao et al. (2016) iNEXT package.

Using a multivariate approach, we explored and described our data using a three‐step procedure, taking into account the type of variables (quantitative or qualitative) considered. As quantitative variables, we determined (i) species abundances (i.e. number of individuals trapped) by trap, room, locality and group of localities, and (ii) capture rates (i.e. number of individuals of a given species divided by trapping effort) for each locality. As qualitative variables, we considered the type of trap (wire mesh or Sherman) at the trap scale, and the presence of food, the type of room and nature of the floor (mostly concrete vs. clay, aka “banco”), walls (mostly concrete vs. clay) and ceiling (mostly concrete or corrugated iron vs. straw) at the room scale. Note that variables noted at the scale of the rooms were aggregated in percentages for each locality. We first performed (i) a centred principal component analysis (cPCA) on the localities × species table (using square roots of trapping success as data); (ii) a fuzzy correspondence analysis (fCA), on the localities × room characteristics (using numbers of each modality for each variable); and (iii) a *K* + 1 analysis coupling the previous two analyses (Bougeard & Dray, 2018; Bougeard et al., 2011), with the aim of describing the relationships between these two types of data (rooms treated through partial least squares (PLS) regressions and mammals described through a cPCA). The method is a multiblock PLS regression (mbpls) applied to the particular case of a single‐response dataset. Block of response variables are explained by a large number of explanatory variables which are divided into *K* meaningful blocks. All the variables – explanatory and dependent – are measured on the same localities. The main results are summarized using overall graphical displays. All data were analysed using ade4 R package (Chessel et al., 2004; Dray et al., 2007; R Core Team, 2022).

Then, a Pearson's Chi‐squared test was realized on the contingency table enumerating the numbers of captures of the seven main species of the small mammal community in the room types recorded, in order to evaluate whether room types may explain the local distribution of each species. The habitat component of the ecological niche of each species was further evaluated using its distribution in the different room types (considered as integrative descriptors of microhabitat) recorded. Following Pianka (1973), we used two indices to characterize each species niche and their overlap between species pairs:

1. Niche breadth quantified using Simpson's index of diversity *B* = $1/\sum p_{i}^{2}$, where *p*_*i*_ is the proportion of the *i*th room type actually used by the species.
2. Niche overlap based upon Levin's (1968) index O_*ij*_ = ∑*p*_*ij*_ *p*_*ik*_/√($\sum p_{\mathrm{ij}}^{2}\sum p_{\mathrm{ik}}^{2}$), where *p*_*ij*_ and *p*_*ik*_ are the proportions of the *i*th room type used by the *j*th and the *k*th species, respectively

Finally, we examined co‐occurrence patterns through the analyses of presence–absence matrices with “null model” randomization tests of marginal row and column totals (Gotelli, 2000; Gotelli & Ulrich, 2010) using *pairs* software (Ulrich, 2008). Aggregated/random/segregated pattern of co‐occurrence of species pairs was inferred from the *p* value associated with the *Z*‐score for each pair of species, either using the global dataset (from all 49 localities) or local datasets (per locality and per district in large cities). We used the “fixed row–fixed column” and “fixed row–equiprobable column” randomization algorithms to generate randomized matrices that serve as null models as advised by Gotelli (2000), and ran the models with 10,000 iterations.

### 2.4 Ethical statement

Permission to enter and work within villages was systematically obtained from the appropriate institutional, traditional and familial authorities. Trapping sessions were carried out in accordance with requirements of Senegalese and French legislations. Every protocol used here received prior explicit approval from the relevant institutional committee (Centre de Biologie pour la Gestion des Populations (CBGP): *Agrément pour l'utilisation d'animaux à des fins scientifiques* E 34‐169‐001). All animal‐related procedures were performed according to official ethical guidelines provided by the American Society of Mammalogists (Sikes & Gannon, 2011). Euthanasia of less than 200 g specimens was performed via cervical dislocation as recommended by Mills et al. (1995), with previous parenteral injection of a derivative of pentobarbital in larger individuals (*Cricetomys gambianus* especially), as recommended by AVMA (2020).

## 3 RESULTS

### 3.1 Trapping results

The total trapping effort represented 13,283 trapnights, which led to the capture of 3160 small mammals, including 2590 rodents, 569 shrews (g. *Crocidura*) and 1 hedgehog (*Atelerix albiventris*; Table 1). Regarding the specific abundance, exotic species were dominant, with first of all *M. musculus* (*N* = 945 captures, 30% of the total captures), then *R. rattus* (*N* = 804, 25%). The native shrew *Crocidura olivieri* (*N* = 556, 18%), the two species of *Mastomys* (*M. natalensis*, *N* = 308, 10%; *M. erythroleucus*, *N* = 278, 9%), *Arvicanthis niloticus* (*N* = 141, 4%) and *Praomys daltoni* (*N* = 91, 3%), followed. The remaining individuals, determined as *Atelerix albiventris*, *Cricetomys gambianus*, *Crocidura* sp., *Gerbilliscus gambianus*, *Mastomys* sp., *Mus* (including the subgenus *Nannomys*) spp. and *Steatomys* sp. accounted for ca. 1.2% of the total captures (*N* = 37). Figure 1 presents the relative frequencies of these species per locality. Regarding their geographic distribution, the species present in the largest number of localities were, respectively, *C. olivieri* (*N* = 39 localities) and *M. erythroleucus* (*N* = 33), followed by *R. rattus* (*N* = 29), *P. daltoni* (*N* = 24), *M. musculus* (*N* = 21) and *A. niloticus* (*N* = 18). At the same time, the exotic rodents *M. musculus* (in *N* = 16 localities) and *R. rattus* (*N* = 14) were the species more often dominant numerically, far ahead of *C. olivieri* (*N* = 7) and *M. natalensis* (*N* = 6).

*Mus musculus* is known to be badly sampled by the wire‐mesh traps we use (young/small individuals can escape from the traps whose mesh is too big compared to their size), but very well sampled by Sherman traps. The reverse tends to be true for *Rattus rattus* (Granjon et al., 2021). Knowing that, we generally use roughly the same number of traps of the two types in all localities, and place one trap of each type at close proximity in each room sampled to limit potential sampling biases. As a result, Sherman traps represent 49.2% of the traps set in the 21 localities harbouring *Mus musculus*. We nevertheless tested the relation between *Mus musculus* abundance and the trapping effort with Sherman traps (that ranged between 40.3% and 51.1%) in these 21 localities. This relation appeared to be only weakly and non‐significantly positive (coefficient of determination of the linear regression *R* ^2^ = .17, *p* = .063, df 19), suggesting that these small differences in trapping effort with Sherman traps between localities may not have entailed major biases in species abundance estimates.

Diversity analyses produced rarefaction curves (tending to an asymptote) and completeness curves (reaching 1) characteristics of well‐sampled assemblages, at both the locality and area scale (Appendix 2). This was the case even when trapping effort (in terms of number of nights or number of trapnights) was quite small (2 nights and/or around 100 trapnights, such as Birkelane, Panal or Yaféra), or in areas represented by only three to four localities (Petite Cote, Basse Casamance and Haute Casamance).

### 3.2 Community structuration

The corresponding data (limited to the seven most captured small mammal species) were subjected to cPCA at the scale of the nine areas encompassing the 49 localities; the first axis of which (Appendix 3) showed distinct distribution trends for the exotic *M. musculus* and all other small mammals. *Mus musculus* appears to be highly dominant overall in the coastal area North of the Gambia as well as on the Kaolack–Tambacounda axis, and present at high frequency in Tambacounda, in localities around Kidira (at the Senegal–Mali border) and in Haute–Casamance. Conversely, this species is absent from Basse–Casamance and south‐eastern Senegal, where the exotic *R. rattus* or native species mainly occur (Figure 1). The second axis of the cPCA mostly showed a contrasted distribution of both exotic species and *C. olivieri* versus the native *M. natalensis* which is largely dominant in the Kédougou region, and is limited in the west by the eastern limit of Niokolo‐Koba National Park, and the locality of Bransan (n°7 in Figure 1) to the north. The other native rodent species (*M. erythroleucus*, *P. daltoni* and *A. niloticus* in particular) are generally present in low frequency at all localities. However, we can note their particularly high proportions in villages of the Senegal river Valley north of Bakel (locality n°1 in Figure 1). *Crocidura olivieri* is rarely absent from sampled localities, and regularly (co)dominant in catches around Tambacounda and on both sides of the Tambacounda–Kidira axis.

### 3.3 Habitat preferences

**FIGURE 2:**
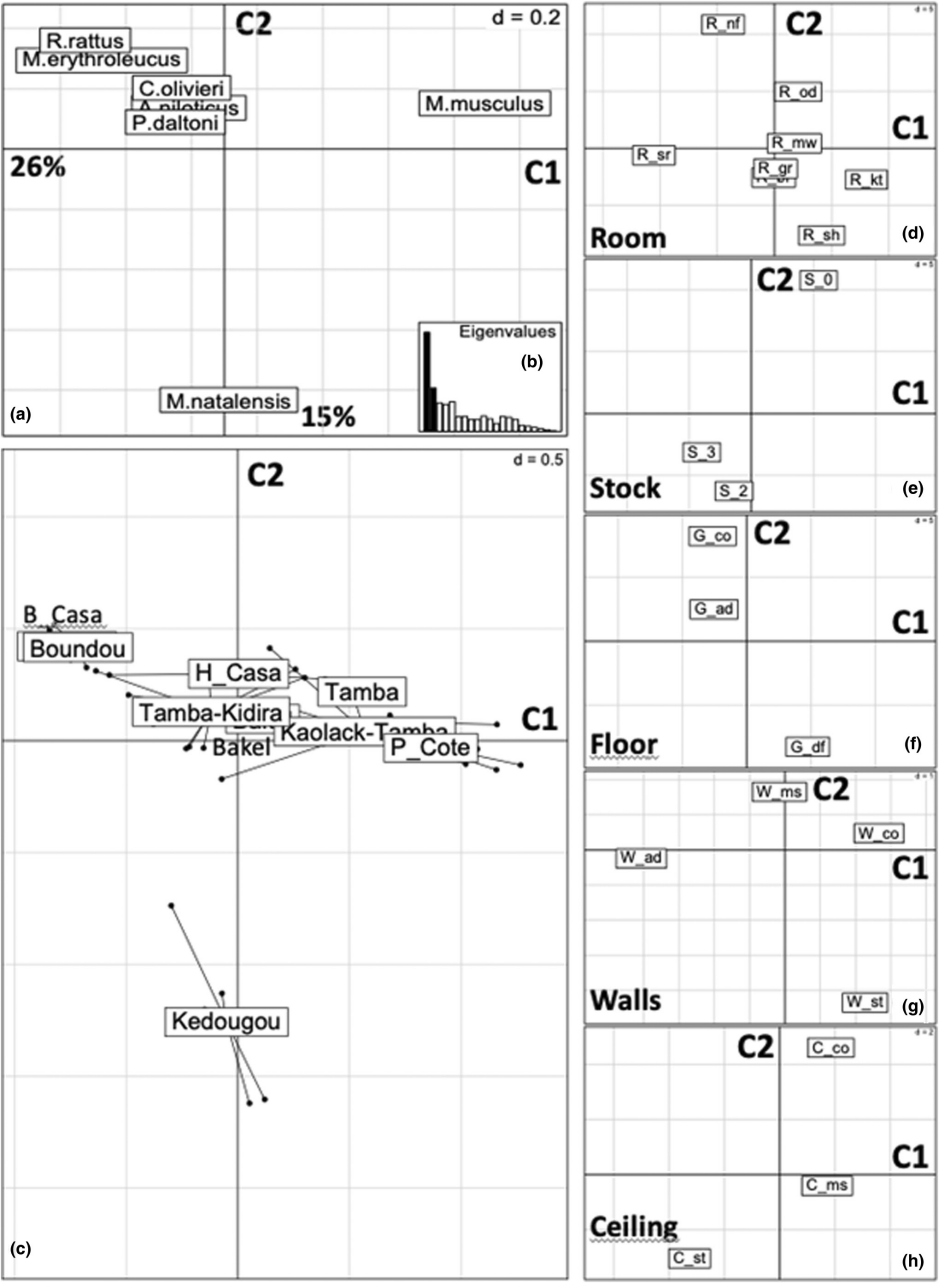
*k* + 1 analysis of the room characteristics and the rodent communities. (a) C1–C2 factorial map of the rodents. (b) Eigenvalues graph of the *k* + 1 analysis. (c) C1–C2 Factorial map of the localities grouped by areas. (d–h) C1–C2 factorial maps of the room characteristics; (d) Room type (br, bedrooms; gr, granaries; kt, kitchens; mw, workshops; nf, non‐food stores; od, outdoors; sh, food shops; sr, stock rooms); (e) Stock (0: no stock, 2: some stock present, 3: large stock present); (f) Floor (ad, adobe; co, concrete; df, dirt floor); (g) Walls (co, concrete; ms, metal sheet; mw, mud wall; st, straw or without); (h) Ceiling (co, concrete; ms, metal sheet; st, straw, adobe or without).

The distribution of all the captures of the seven main species of the community in the eight room types is given in Table 2. Small mammal species appear not to be randomly distributed in the room categories defined (khi‐2 = 401.34; 42df; *p* = 3.62 × 10^−60^). The distribution of *M. musculus* appears as the most divergent from random expectations, the species being clearly over‐represented in kitchens (and to a lesser extent in stores) and under‐represented in outdoors, granaries and stock rooms. The distribution of the room types and their modalities (nature of floor, walls and ceiling) across the localities sampled did not show any particular trend, as evidenced by the results of fCA (Appendix 4). An overall opposition between more urbanized (Petite Côte, Tambacounda) versus more rural (Boundou) areas appears, however, associated with a dominance of distinct room type (workshops and non‐food stores vs. granaries) or construction materials (concrete and metal vs. adobe and straw). From there, a *k* + 1 analysis was performed between the cPCA of small mammals and the fCA of room types/modalities grouped by geographic areas. This *K* + 1 C1‐C2 factorial map illustrates graphically (Figure 2) the relationships between species, geographic areas and room types and characteristics. The most visible associations are, on axis 1, between *M. musculus*, kitchens and concrete walls, mainly on the Petite Côte, the Kaolack–Tambacounda axis, and in Tambacounda (positive side of C1). On the opposite (negative) side of C1 are all the other small mammals, *M. natalensis* excepted, stock rooms and adobe walls (and to a lesser extent presence of large food stocks and straw ceiling), in Boundou, Basse Casamance and the Tambacounda–Kidira axis, to a lesser extent. C2 mainly contrasts *M. natalensis* associated with average levels of food and concrete floor in the area of Kédougou versus *R. rattus* and *M. erythroleucus* related to non‐food stores and outdoors, absence of stocks, concrete floor and ceiling and metal sheet walls.

**TABLE 2:** Distribution of the seven main commensal small mammal species within the eight room types defined (between brackets expected numbers under the hypothesis of independence of the two variables).

	*Arvicanthis niloticus*	*Crocidura olivieri*	*Mastomys erythroleucus*	*Mastomys natalensis*	*Mus musculus*	*Praomys daltoni*	*Rattus rattus*	Total
Non‐food stores	0 (4.4)	11 (17.4)	13 (8.7)	0 (9.7)	49 (29.7)	1 (2.9)	24 (25.2)	98
Food Stores	7 (9.3)	32 (36.7)	13 (18.3)	49 (20.3)	75 (62.3)	3 (6.0)	27 (53.0)	206
Bedrooms	23 (51.2)	189 (202.1)	106 (101.3)	133 (111.9)	374 (343.4)	47 (33.1)	263 (292.2)	1135
Kitchens	23 (12.9)	42 (50.9)	5 (25.5)	21 (28.2)	135 (86.5)	7 (8.3)	53 (73.6)	286
Outdoors	15 (6.4)	37 (25.3)	10 (12.6)	2 (14.0)	49 (43.0)	3 (4.1)	26 (36.6)	142
Granaries	18 (6.2)	26 (24.4)	24 (12.2)	4 (13.5)	10 (41.5)	7 (4.0)	48 (35.3)	137
Stock Rooms	55 (48.7)	208 (192.1)	107 (96.0)	99 (106.4)	234 (326.5)	23 (31.4)	353 (277.8)	1079
Workshops	0 (1.8)	11 (7.1)	0 (3.6)	0 (3.9)	19 (12.1)	0 (1.2)	10 (10.3)	40
Total	141	556	278	308	945	91	804	3123

### 3.4 Niche breadth and overlap

Niche breadth and niche overlap values are presented in Table 3. They did not show any differences between exotic and native species. Niche breadth ranges between 2.9 for *Praomys daltoni* and 4.2 for *A. niloticus*. Niche breadth of the domestic mouse is higher than that of the black rat (4 vs. 3.2). Niche overlap values are generally high, ranging between 0.74 and 0.99. *Arvicanthis niloticus* shows the lower mean value (0.84) and *C. olivieri* the highest (0.95). Mean niche overlap values of *M. musculus* and *R. rattus* with other species are the same and are high (0.94), suggesting regular co‐occurrence of these invasive species with native ones at the room scale (see hereunder).

**TABLE 3:** Niche breadth (numbers in italics, on the diagonal) and niche overlap (numbers above the diagonal) of the seven main species.

	*Arvicanthis niloticus*	*Crocidura olivieri*	*Mastomys erythroleucus*	*Mastomys natalensis*	*Mus musculus*	*Praomys daltoni*	*Rattus rattus*
*Arvicanthis niloticus*	*4.2*	0.91	0.86	0.79	0.80	0.74	0.92
*Crocidura olivieri*		*3.7*	0.99	0.96	0.95	0.93	0.99
*Mastomys erythroleucus*			*3.3*	0.95	0.93	0.94	0.98
*Mastomys natalensis*				*3.1*	0.97	0.95	0.93
*Mus musculus*					*4.0*	0.97	0.91
*Praomys daltoni*						*2.9*	0.89
*Rattus rattus*							*3.2*

### 3.5 Co‐occurrence analyses

At the global level (i.e. with localities as sites), two significant patterns of aggregation were revealed, both implying *M. erythroleucus*: with *C. olivieri* (*Z* = −2.20, *p* = .0274 with “fixed row–fixed column” [ff] randomization; *Z* = −2.32, *p* = .0202 with “fixed row‐equiprobable column” [fe] randomization) and with *P. daltoni* (*Z* = −2.01, *p* = .0440 with “fe” randomization). Conversely, five segregation patterns were found significant using at least one of the two randomization schemes. Four of them are suspected to be biased by overall differences in spatial distribution of the species involved, at the scale of southern Senegal: between *M. musculus* and *P. daltoni*, between *C. olivieri* and *M. natalensis*, between *R. rattus* and *M. natalensis* and even more conspicuously between *M. musculus* and *M. natalensis*, which distributions were completely disjoint at the time of sampling (see Figure 1). The last one, implying *M. erythroleucus* and *M. musculus* (*Z* = 3.35, *p* = .00078285 with “ff” randomization, *Z* = 2.84, *p* = .0044 with “fe” randomization), is less prone to being influenced by distribution range differences.

At the locality level (i.e. with “houses” as sites within the 49 localities and districts of Kédougou and Tambacounda, see details in Appendix 5), a total of 586 species pair associations were tested. Only 39 (27 with “fe” + 12 with “ff” randomization schemes, respectively) of them (10.75%) proved to show a significant pattern of segregation or aggregation, 33 (22 + 11) of which concerned the seven most abundant species. They are detailed in Table 4. *Mus musculus* and *R. rattus* were involved in most of the segregation cases (13/15). In the large cities (where more than 50 sites were considered in co‐occurrence analyses), a significant segregation was observed between *M. musculus* and *R. rattus* in Tambacounda as well as in two of its districts, and between *M. natalensis* and both *R. rattus* and *C. olivieri* in Kédougou (where *M. musculus* is absent). Conversely, *M. musculus* was never involved in aggregative patterns, to the opposite of *R. rattus* which was regularly found more often than expected to co‐occur with *C. olivieri* and *M. erythroleucus* (for instance, in Kédougou and Rufisque). Native species of small mammals also show aggregative patterns in a number of other localities.

**TABLE 4:** Co‐occurrence patterns between the seven most common commensal species of small mammals in southern Senegal: localities where significant *Z* values were found for aggregation (above the diagonal) or segregation (below the diagonal) are indicated.

		Aggregation						
		*Mus musculus*	*Rattus rattus*	*Crocidura olivieri*	*Mastomys natalensis*	*Mastomys erythroleucus*	*Arvicanthis niloticus*	*Praomys daltoni*
Segregation	*Mus musculus*			1 district of Tambacounda (ff)				
	*Rattus rattus*	**Tambacounda (fe)** 1 district of Tamba. (fe) 1 district of Tamba. (fe/ff)		Dieylani (fe) **Kédougou (fe/ff) Rufisque (ff)**		Dieylani (fe) **Kédougou (fe/ff)** 1 district of Kédougou (fe/ff) 1 district of Tamba. (fe)		
	*Crocidura olivieri*	1 district of Tambacounda (fe)	Diattacounda (fe) Vélingara (fe/ff)			Dieylani (fe) Diattacounda (ff) Panal (fe)		Bransan (fe)
	*Mastomys natalensis*		**Kédougou (fe)**	**Kédougou (fe)**				
	*Mastomys erythroleucus*		Goumbayel (fe)				Kidira (fe)	Badi‐Nieriko (ff) Bransan (fe) Soutouta (ff)
	*Arvicanthis niloticus*		Soutouta (fe/ff)					
	*Praomys daltoni*			Sémé (fe)				

*Note*: fe, “fixed row‐equiprobable column” and ff, “fixed row‐fixed column” randomization schemes, respectively; in bold those localities where ≥50 sites were sampled; Tamba, Tambacounda.

## 4 DISCUSSION

### 4.1 Commensal small mammal community composition and distribution

A previous analysis of commensal small mammal communities at the scale of Senegal has been presented by Dalecky et al. (2015). While it was primarily focused on the house mouse distribution, this work depicted data gathered between 1983 and early 2014 on the expansion of both exotic *M. musculus* and *R. rattus* versus all native species taken as a whole. The present study extends the effort of Dalecky et al. (2015) both temporally (to September 2015) and spatially by adding large localities such as Tambacounda and Rufisque, and new areas such as the one north of Kidira at the Senegal–Mauritania eastern border. Using data from 49 localities in the southern half of the country (i.e. covering the Senegalese distribution of *R. rattus*), it also details the patterns of occurrence/co‐occurrence of all the small mammal species encountered.

Commensal species can be classified precisely following Hulme‐Beaman et al. (2016) who provided a series of definitions concerning the type of relationship that species can have with anthropogenic environments. According to this terminology, we have here a mixture of “obligate commensals” represented by the exotic invasive species *M. musculus* and *R. rattus* that can only survive in the study area because of their ability to occupy houses, and of “occasional commensals” that occur both within houses and in outdoor habitats (all the native species). Among the latter, *M. natalensis* tends, however, to be an obligate commensal in Senegal (Duplantier & Granjon, 1988), even if the species is known to occur outdoors elsewhere in Africa (Leirs, 2013).

In the sample gathered here, exotic invasive species outnumbered native ones from nearly all points of view. Indeed, *M. musculus* and *R. rattus* represented more than 55% of all the small mammals captured (1749/3160). They were also found dominant in the largest number of localities (16 and 14, respectively), even though they are not present in the majority of them. These data testify for the success of these invasive species in Senegal, where the trend towards a rapid west‐to‐east expansion (i.e. from coastal areas where they were first introduced, to inland) has been spectacular over the last decades (see fig. 1 in Dalecky et al., 2015). In other words, once these species colonize a new place, they can rapidly become dominant over native ones. This seems to be especially the case for *M. musculus* which is dominant in the majority of the localities where it is present (16/21). This potential to rapidly invade a small mammal community and extirpate the native species previously present has been documented in a number of localities of northern Senegal over the last two decades (Dalecky et al., 2015; Diagne et al., 2020, 2021). It even seems that this recent expansion of *M. musculus* has come at the expense of *R. rattus*, as suggested by the comparison of the data presented in Duplantier et al. (1991) and ours. One may bet that this situation of dominance of invasive species over native ones in commensal small mammal communities is going to become the rule in a number of regions/countries all over Africa. As an example, *R. rattus* appears as often dominant in Benin's localities in the survey of Hima et al. (2019) along a Benin–Niger axis. In Guinea, *M. musculus* was only found in the coastal region by Demby et al. (2001), especially in the city of Kindia where its abundance decreased from the centre to the periphery. Later, Fichet‐Calvet et al. (2005) found *R. rattus* as the dominant commensal species in smaller villages of the coastal region. Interestingly, nearly 30 years ago, the study by Olaseha et al. (1994) suggested that *Rattus* spp. and *M. musculus* were already the main commensal species in the urban and rural areas they studied in south‐western Nigeria. This may also be the trend in the New World where, even in rural areas, invasive rodents already constitute the bulk of small mammals found within houses. For instance, 74% of the rodents caught in various urban habitats of the city of Rio Cuarto, province of Cordoba, Argentina (Castillo et al., 2003), were of (mainly) *M. musculus* and *Rattus* spp.; similarly, 92% of the captures indoors and in the yards surrounding the houses were of *M. musculus* and *R. rattus* in a rural area of Yucatan State, Mexico (Panti‐May et al., 2012).

The only native rodent species that stays dominant wherever present is *M. natalensis*. However, it has to be underlined that this species, restricted to the south‐eastern part of Senegal (Duplantier & Granjon, 1988), is only co‐occurring with an exotic invasive species (here *R. rattus*) in one locality, namely Kédougou; this locality constitutes the invasion front of the species in this part of the country. There, *M. natalensis* has apparently resisted the arrival of *R. rattus*, which occurred at the end of the 1990s (Bâ, 2002), since its dominance in this city has continued until this period (unpublished data). This situation also occurs in villages of Upper Guinea where *M. natalensis* was found as the main commensal species (Fichet‐Calvet et al., 2009), as well as in a number of localities of Niger, including the majority of its capital city (Niamey) districts (Garba et al., 2014; Hima et al., 2019).

In our dataset, the other native rodent species which stay dominant in only a small number of localities are *M. erythroleucus* and *P. daltoni* in the extreme East of the study area, where *M. musculus* is apparently progressing and is expected to replace them in a near future. As found by Hima et al. (2019) at lower latitudes, the native small mammal species which finally stays as the most regularly present, and often co‐dominant with invasive rodents, is the shrew *C. olivieri*. This species proves here that it can behave as a true commensal species even if rarely presented as such (Churchfield & Hutterer, 2013).

### 4.2 Habitat preferences and niche breadth/overlap

Habitat represents one of the main niche dimensions, which has often been considered in community ecology studies (Morris, 1996). Nevertheless, most studies conducted to date concerned communities in outdoor environments, where habitats may differ according to several factors like vegetation, soil and elevation, among others. Here, room type and rooms characteristics were chosen as easy‐to‐describe proxies of habitat/microhabitat structure that may be relevant for commensal small mammals. Indeed, the variables recorded here enable us to distinguish between categories of domestic spaces, in terms of “privacy” (from bedrooms to outdoor spaces or shops), type of activities hosted, hiding places and food resources present. Also, the nature of construction materials used for rooms can help to distinguish between traditional (use of clay for floor and walls, and of straw for ceiling) and more modern buildings (use of concrete for floor and walls, and of corrugated iron for ceiling), the latter being expected in villages that are more integrated into commercial networks and directly connected to large cities, thus more prone to the introduction of exotic rodents (Diagne et al., 2016, but see Lucaccioni et al., 2016).

Our analyses present *M. musculus* as more abundant in some room types (kitchens and stores), especially when built with non‐traditional construction materials (cement and iron, particularly), similarly to *M. natalensis* in its area of occurrence. These habitat types contrast with those where *A. niloticus* is found more often than expected (in granaries and outdoors), which is coherent with the ecology of the latter species, more abundant in grassy habitats and grain fields in outdoor environments (Granjon et al., 2013). *Arvicanthis niloticus* also shows both the larger niche breadth and the lower mean overlap with other species. These characteristics may represent attributes of the “occasional commensal” category of Hulme‐Beaman et al. (2016), of which *A. niloticus* is probably the most extreme representative. Interestingly, *P. daltoni*, which is regularly found indoors in West Africa (Brýjá et al., 2010), has the smallest niche breadth – being often under‐represented in the room types sampled, and nearly only over‐represented in bedrooms, mainly in the extreme East of the country. This species may suffer from the arrival of exotic invasive species and be pushed back into the innermost rooms of the houses until it is excluded. The only non‐rodent species, that is, the shrew *C. olivieri* appears as very catholic in its habitat preference, being found in all room types in numbers close to those expected from their proportions in the overall sample. This also translates into a relatively high value of niche breadth, and also high niche overlap values with all rodent species. The wide range of habitats occupied and adaptability of this species have already been underlined (Churchfield & Hutterer, 2013). The habitat niche overlap with rodent species here observed probably relates to the fact that this shrew does not belong to the same ecological guild (sensu Simberloff & Dayan, 1991) and, as such, is probably not submitted to competitive interactions with them likely to constrain its ecological distribution. Here, another type of interaction may rather be at work between shrews and rodents, namely a predator–prey relation: a preliminary metabarcoding study of the gut and faeces content of *C. olivi*eri individuals provides support for such a hypothesis that would imply active predation, possibly mostly on neonates or non‐active unweaned juveniles, directed primarily against *M. musculus* (Galan et al., 2023). *Rattus rattus*, which was very abundant in stock rooms where it probably causes important damage to food stuff (see Dossou et al., 2020, for an example in Cotonou, Benin), presents an average value of niche breadth compared to other species, and high overlap values with other species. Using telemetry in a rural area around Berega in Tanzania, Monadjem et al. (2011) found that within the houses or buildings they live in, black rats (also called roof rats) were located in the roof (37% of fixes), in the bedroom (35%), kitchen (14%) and in walls and windows (14%). Even if some *R. rattus* were caught in traps set on top of furniture items or wall tops, we were not able to quantify the three‐dimensional activity of the species known to be at home in the upper parts of dwellings (Granjon & Duplantier, 2009; Monadjem et al., 2011). This vertical component of its spatial niche may, however, participate in the ecological distribution of the species and help its coexistence with the other ones. Its tolerance for quite traditional and rural conditions also makes it a good candidate for long‐term persistence in relatively marginal areas, even in the absence of intense and regular road traffic (Lucaccioni et al., 2016).

As precised by Colwell and Futuyma (1971), such raw measures of actual niche breadth and overlap cannot per se give conclusive answers on the potential competition between coexisting species from a community. However, they can help formulate hypotheses to be tested via experimental procedures. In between, co‐occurrence analyses may also help go further in the understanding of actual interspecific relationships at various spatial scales.

### 4.3 Co‐occurrence patterns

Co‐occurrence analyses at large geographic scales give information on patterns issued from historical processes, often shaped by life‐history traits of the species involved (see Davis et al., 2018 for an example on Carnivores). Concerning West African commensal rodents, the only significant interspecific segregation pattern found by Hima et al. (2019) among the four dominant species (*M. natalensis*, *R. rattus*, *Crocidura* spp. and *R. norvegicus*) along the Cotonou (Benin)–Niamey (Niger) corridor was between *R. norvegicus* and *M. natalensis*. Conversely, the two *Rattus* species and the pair *R. norvegicus*/*Crocidura* spp. showed significant aggregation at this spatial scale (i.e. they were found more often than expected by chance in the same localities). The authors did not propose any explanation of these trends, which may typically result from a mixture of historic and stochastic processes on the one hand, and behavioural ones on the other hand, especially when the co‐occurrence event does correspond to real co‐existence/syntopy on the microhabitat scale. In Senegal, Dalecky et al. (2015) showed that aggregative patterns between native species of rodents seem to be disrupted by the presence of *Mus musculus* in commensal assemblages. At the scale of the city of Niamey and using different methodological approaches, Garba et al. (2014) found strong segregation patterns between native *M. natalensis* and both invasive *R. rattus* and *M. musculus*, whereas the latter two species showed either random or slightly aggregated (depending on the set of districts considered) co‐occurrence patterns. Invasive rats and mice were found associated with urban areas characterized by intense commercial and exchange activities (markets, coach stations and stores) that lie in the heart of town. In these habitats, they probably replaced native *M. natalensis* which has been formerly present, leading to the native/invasive segregation patterns observed.

Here, we were able to tackle the species co‐occurrence questions at two different scales, thanks to our standardized sampling protocol. At the global scale, aggregation cases were only observed between native species that probably share the commensal space for long. Both cases involved *M. erythroleucus*, with a relatively closely related rodent species (*P. daltoni*) on the one hand, and with the shrew (*C. olivieri*) on the other hand. Interestingly, these three species can be considered as the most prone to live as commensals of humans among native ones, with the exception of *M. natalensis*, which may partly explain their regular associations in the localities sampled. Most of the segregation patterns observed at this scale cannot be discussed as they are likely biased by distribution differences between the species concerned. Conversely, the segregation observed between the invasive *M. musculus* and the native *M. erythroleucus*, well supported using both randomization schemes, is especially interesting as it echoes the situation observed in northern Senegal where the house mouse is progressively, and apparently rapidly, replacing native rodents (and especially *M. erythroleucus*; Dalecky et al., 2015; Diagne et al., 2021). The processes underlying this invasion success are not yet fully understood, but they may include parasitological (Diagne et al., 2016, 2020, 2021) and/or immunological (Diagne et al., 2017) aspects. The speed of this replacement, which was estimated to cover a few dozen years by Dalecky et al. (2015), is here highlighted by the segregation pattern observed, which tends to indicate that once the house mouse has colonized a new locality, *M. erythroleucus* rapidly declines in abundance, until it disappears. New samplings in sites where *M. erythroleucus* was still present in this 2013–2015 time window, especially along the Tambacounda–Kidira axis and along the Mauritania–Senegal border, would confirm this trend if it showed that the house mouse had become the dominant, or even the unique, rodent species present.

At the local scale, various patterns were observed between the commensal species in southern Senegal. The segregation observed in Tambacounda as well as in some of its districts between the invasive *R. rattus* and *M. musculus* was among the most significant. This trend towards a mutually exclusive distribution in separate housing or working plots may be the result of direct or indirect interactions between these two species. Such interactions have been documented in outdoor habitats of Pacific islands as in the Galapagos or New‐Zealand (Bridgman et al., 2018; Harper & Cabrera, 2010). In these cases, a negative impact of *R. rattus* on *M. musculus* was suspected, based more on indirect (risk effect) than direct / exploitation competition (possibly including predation by *R. rattus* on *M. musculus*). The processes at work in complex commensal environments such as those found in large cities may be different, and the outcome of the interactions may not systematically benefit the larger species (here the black rat). Instead, the house mouse may well be favoured in urbanized environments such as those that are developing in sub‐Saharan Africa, as exemplified by the situation observed in Dakar (Stragier et al., 2020), and in most of the cities from the western part of Senegal North of the Gambia, that is, the area which has benefitted from the groundnut trade for its early and accelerated development since the 1960s (Lombard et al., 2020). In such habitats, the small size of the house mouse could represent a real advantage to (i) better hide from predators (including humans), (ii) more easily slip into well‐protected buildings and rooms and (iii) subsist on less abundant food resources. From there, competition with larger rodent species (including native ones) may not represent a hindrance to the house mouse range expansion, contrary to what has been hypothesized from results obtained on experimental versus control grids in a 150,000‐inhabitant city of central Argentina (Gomez et al., 2008). The continuous development of urbanization according to modern standards along the West‐to‐East major communication axes (mainly roads) should therefore lead to the continued invasion of the country by *M. musculus*, a trend that could be confirmed in the future by re‐sampling localities where the species is either absent or sharing the space with *R. rattus*.

Other major cases of segregation at the local scale involve *M. natalensis* in Kédougou, with both *C. olivieri* and *R. rattus* (only with one randomization scheme, however). Here also, interactions probably occur regularly between these species that appear abundant in this city, which may have led to some kind of mutual exclusion at the scale of the housing or working units sampled. Competition between *M. natalensis* and *R. rattus* is regularly proposed to be at work, or to have occurred in situations where they were confronted: in Eastern RDC villages, it turned to the advantage of the black rat that replaced *M. natalensis* in a number of villages during the first half of the 20th century (Misonne, 1959). In Tanzania and Swaziland, the fact that *M. natalensis* rarely entered houses was associated with the dissuasive presence of *R. rattus* (or *R. tanezumi*) in this habitat, a hypothesis which was strengthened by the regular observation of *M. natalensis* in commensal habitat in Namibia where no *Rattus* species occurs (Monadjem et al., 2011). Trying to find out which process may underlie this potential exclusion of *M. natalensis* by *R. rattus* in commensal habitats, Cuypers et al. (2017) failed to demonstrate an avoidance behaviour mediated by scent markings. Additional work is necessary to understand the processes at work, but the relative stability of the ratio *R. rattus*/*M. natalensis* in Kédougou since the arrival of the former species in this city more than 25 years ago (Bâ, 2002) advocates for good competitive skills of *M. natalensis* in this context. This is all the more apparent as, immediately around the distribution area of *M. natalensis* in southern Senegal, the black rat is very well installed and often dominant (Dalecky et al., 2015; Duplantier et al., 1997; Lucaccioni et al., 2016; this study).

At the same time, it has to be noticed that *R. rattus* and *C. olivieri*, in Kédougou as in other localities (namely Rufisque and Dielyani), show a clear aggregative pattern, suggesting that they apparently cohabit quite easily in the commensal space. The fact that the black rat partly forages and lives in upper parts of buildings while the shrew exclusively lives at ground level may explain such cohabitation. These two species were also the most regularly involved in aggregative associations with native rodent species, and especially with *M. erythroleucus*. This may testify to an ancient cohabitation history between these species (more ancient than with *M. musculus*, in particular), and/or be linked with less overall niche overlap between them. The latter is not apparent when looking only at the microhabitat dimension, but may involve dietary, space use or other niche components.

## 5 CONCLUSIONS

We here present a “snapshot” picture of the community structure of commensal small mammals captured in southern Senegal. This area corresponds to the current distribution area of *R. rattus*, a major invasive species well established for more than one century in this part of Senegal (Konečný et al., 2013). Most of this area has apparently been colonized more recently by *M. musculus*, another major invasive rodent species with rapid and ongoing invasion dynamics (Dalecky et al., 2015; Lippens et al., 2017). The contact between these invasive species and the native ones may therefore date from various periods according to the time of arrival/installation of *R. rattus* and *M. musculus*. This probably results in communities that cannot be considered at equilibrium in a number of cases, which in turn makes it difficult to envisage stabilized assembly rules in these species assemblages (see also Hima et al., 2019).

Nevertheless, the results obtained here, associated with others presented recently on each of these two invasive species in Senegal (e.g. in Diagne et al., 2021; Lucaccioni et al., 2016; Stragier et al., 2020), help to better understand their ecological characteristics and requirements, and to make some hypotheses on the evolution of the communities they constitute with their native counterparts in commensal contexts. Indeed, the invasive black rat and house mouse do not seem to have very specific habitat requirements, and they share similar niche breadth with native species in this respect. They also show important overlap in terms of room types they occupy, which should lead to frequent interactions. Other components of the ecological niche of these species should be considered, which may be more informative on the outcome of co‐occurrence patterns and interspecific interactions. In these communities where the spatial range dynamics of the invasive species are rather well known, a better knowledge of both niche characteristics and the nature of interactions between the species concerned will enable us to better understand co‐occurrence patterns, and even to make some predictions on the temporal evolution of these patterns at different spatial scales (Bar‐Massada, 2015). At the local scale, fine‐grained co‐existence mechanisms would worth be studying in large cities showing both habitat complexity and a reasonable diversity of interacting species (such as Kédougou or Tambacounda). In addition to continuous spatio‐temporal surveys over the studies areas to capture the changing dynamics within these small mammal communities, further multidisciplinary research efforts should be devoted to (i) unravelling the multifactorial mechanisms underlying the (potential) changes observed in the community structure over time, (ii) depict the consequences of these modifications at ecological (e.g. species extirpation), social (e.g. threats to stored food) and/or health (e.g. emergence of rodent‐borne zoonoses) levels, and (iii) move – by concerted efforts with local stakeholders and decision‐makers – from fundamental empirical results to sustainable and efficient management actions against the detrimental effects of some of these small mammals.

## AUTHOR CONTRIBUTIONS

**Laurent Granjon:** Conceptualization (equal); formal analysis (equal); investigation (equal); supervision (equal); writing – original draft (lead); writing – review and editing (equal). **Emmanuelle Artige:** Data curation (equal). **Khalilou Bâ:** Investigation (equal). **Carine Brouat:** Funding acquisition (equal); supervision (equal); writing – review and editing (equal). **Ambroise Dalecky:** Investigation (equal). **Christophe Diagne:** Investigation (equal); writing – review and editing (equal). **Mamoudou Diallo:** Investigation (equal). **Odile Fossati‐Gaschignard:** Formal analysis (equal). **Philippe Gauthier:** Investigation (equal). **Mamadou Kane:** Investigation (equal). **Laëtitia Husse:** Investigation (equal). **Youssoupha Niang:** Investigation (equal). **Sylvain Piry:** Data curation (equal). **Nathalie Sarr:** Data curation (equal). **Aliou Sow:** Investigation (equal). **Jean‐Marc Duplantier:** Conceptualization (equal); funding acquisition (equal); investigation (equal); supervision (equal); writing – review and editing (equal).

## CONFLICT OF INTEREST STATEMENT

The authors declare that they have no competing interest regarding this article.

## Data Availability

The dataset on which this paper is based is publicly available on the IRD data repository *DataSuds* (https://doi.org/10.23708/PQTQDA) as a living and updating resource. The static version of this data set is also stored as a supporting information data file in Granjon et al. (2021) (as Commensal_small_mammals_southern_Senegal_2012_2015.csv).

## APPENDIX 1

### 1.1

Schematic map of the localities sampled and the geographic areas within which they were classified (see Table 1 for details).

## APPENDIX 2

### 2.1

Sample size‐based rarefaction and extrapolation sampling curves (left), and sample completeness curves (right) at two sampling scales, namely localities and areas (see text), using Chao et al. (2016) iNEXT Online: Software for Interpolation and Extrapolation of Species Diversity. Program and User's Guide published at http://chao.stat.nthu.edu.tw/wordpress/software_download/inext‐online/.

Rarefaction (left) and completeness (right) curves for 48 of the localities sampled for their small mammal communities (Tambacounda is figured hereunder with the “area” curves).

## APPENDIX 3

### 3.1

Centred principal component analysis (cPCA) of the small mammal capture data in the different areas/localities. (a) C1–C2 factorial map of the species. (b) Eigenvalues graph of the cPCA. (c): C1–C2 factorial map of the localities grouped by areas.

## APPENDIX 4

### 4.1

Fuzzy correspondence analysis (fCA) of the room characteristics per areas/localities. (a) C1–C2 factorial map of the room types (br, bedrooms; gr, granaries; kt, kitchens; mw, workshops; nf, non‐food stores; od, outdoors; sh, food shops; sr, stock rooms). (b) Eigenvalues graph of the fCA. (c–f) C1–C2 factorial maps of the room characteristics (c: Stock. 0: no stock, 2: some stock present, 3: large stock present. (d) Floor. ad, adobe; co, concrete; df, dirt floor. (e) Walls. co, concrete; ms, metal sheet; mw, mud wall; st, straw or without. (f) Ceiling. co, concrete; ms, metal sheet; st, straw, adobe or without). (g) C1–C2 factorial map of the localities grouped by areas.

## APPENDIX 5

### 5.1

Number of sites (localities in the global analysis, housing/working units in the local analyses), number of co‐occurrence tests performed using “pairs.exe” (overall and in each locality) and number of significant tests using the “fixed row ‐ equiprobable column” and the “fixed row – fixed column” randomization scheme. In Kédougou and Tambacounda, results obtained in districts with ⩾30 sites are also presented. Data from Bala, Boutougoufara and Dianké Makha could not be analysed (“Matrix fill too low or too high” error message).

**Table:**

Locality	Number of sites	Number of tests (fe + ff)	Number of significant tests (fe + ff)	Number of significant tests with the seven main species (fe + ff)
All localities (global analysis)	49	156	12 + 13	7 + 7
Badi‐Nieriko	37	12	0 + 1	0 + 1
Bakel	47	6	0	0
Bransan	21	20	2 + 0	2 + 0
Dembankane	22	6	0	0
Diakene‐Wolof	35	6	0	0
Diattacounda	25	12	1 + 1	1 + 1
Diawara	32	12	0	0
Dieylani	29	12	3 + 0	3 + 0
Doulouyabe	23	12	0	0
Fadiouth	21	2	0	0
Gandiaye	13	6	0	0
Goudiry	37	2	0	0
Gouloumbou	27	12	0	0
Goumbayel	17	12	1 + 0	1 + 0
Ida Seco	16	56	4 + 1	0 + 0
Joal	43	2	0	0
Kedougou	144	30	4 + 2	4 + 2
Ked‐Dalaba	30	20	1 + 1	1 + 1
Ked‐Dande Mayo	31	6	0	0
Ked‐Mosquee	45	12	0	0
Kidira	22	30	1 + 0	1 + 0
Kothiary	29	12	0	0
Kounkane	26	20	0	0
Koussan	36	12	0	0
Mako	27	2	0	0
Marsassoum	16	6 + 6	0	0
Méréto	47	20	0	0
Ndiobene	23	2	0	0
Niahene	11	12	0	0
Panal	15	2	1 + 0	1 + 0
Rufisque	50	12	0 + 1	0 + 1
Sabodala	24	6	0	0
Segou	39	2	0	0
Seme	28	12	1 + 0	1 + 0
Sil	22	6	0	0
Sinthian Koundara	29	12	0	0
Sinthiou Doube	10	12	0	0
Sinthiou Maleme	23	2	0	0
Soutouta	45	20	1 + 2	1 + 2
Tambacounda	335	42	2 + 1	1 + 0
Tamba‐Aynina Fall	37	6	1 + 0	1 + 0
Tamba‐Garage K. + Legal Pont	50	6	1 + 2	1 + 2
Tamba‐Marche	46	6	1 + 0	1 + 0
Tamba‐Medina Coura	30	6	0	0
Tamba‐Sare Guilel	33	6	0	0
Tamba‐Sonacos	38	6	1 + 0	1 + 0
Tobor	21	12	0	0
Tuabou	14	12	0	0
Vélingara	29	6	1 + 1	1 + 1
Yafera	12	12	0	0
Youppe Hamady	21	12	0	0
Total (incl. global scale)	1932	742	39 + 25	29 + 18
Total local scale		586	27 + 12	22 + 11
